# Relationship between Cortisol Changes during the Night and Subjective and Objective Sleep Quality in Healthy Older People

**DOI:** 10.3390/ijerph17041264

**Published:** 2020-02-16

**Authors:** Matias M. Pulopulos, Vanesa Hidalgo, Sara Puig-Perez, Teresa Montoliu, Alicia Salvador

**Affiliations:** 1 Department of Experimental Clinical and Health Psychology, Ghent University, 9000 Gent, Belgium; 2 Department of Psychology and Sociology, Area of Psychobiology, University of Zaragoza, 44003 Teruel, Spain; 3 Department of Psychobiology and IDOCAL, University of Valencia, 46010 Valencia, Spain; 4 Area of Health Sciences, Valencian International University, 46002 Valencia, Spain

**Keywords:** sleep quality, subjective sleep, objective sleep, older people, HPA axis, cortisol

## Abstract

The aim of this study was to investigate whether the nighttime cortisol release was associated with subjective and objective sleep quality and the discrepancy between them. Forty-five healthy older adults (age range from 56 to 75 years) collected salivary samples immediately before sleep and immediately after awakening on two consecutive nights. Actigraphy was used to assess objective sleep quality and quantity. A sleep diary was used to assess subjective sleep quality. Linear mixed models were performed using subjective and objective sleep quality data from 76 nights to investigate between-subject associations. We observed that larger changes in cortisol levels between sleep onset and awakening, reflecting a healthier circadian rhythm of the Hypothalamic-Pituitary-Adrenal (HPA) axis, were associated with better subjective sleep quality, but not with objective sleep quality. Moreover, smaller changes in nighttime cortisol were associated with lower subjective sleep quality relative to objective sleep quality. All these results were observed even after controlling for important confounders such as sleep quantity, age, sex, subjective socioeconomic status, stress perception, depression, physical activity, and adherence to the salivary sampling protocol. This study demonstrates that subjective sleep quality in older people may be explained, to some extent, by the activity of the HPA axis.

## 1. Introduction

Normative aging is associated with changes in sleep patterns [1]. Previous research has identified reduced sleep quality as one of the most critical changes in healthy aging. Importantly, reduced sleep quality has been associated with important age-related health problems, such as cognitive deficits, including higher risk of dementia, mood disorders, and cardiovascular disease [2,3,4]. Investigating the factors associated with sleep quality may offer critical evidence to understand, prevent, and treat sleep problems and the negative effects of sleep problems in older people.

Sleep quality is a complex construct, and it can be evaluated both objectively with polysomnography or actigraphy (e.g., sleep efficiency, understood as the percentage of time in bed that an individual is actually sleeping), and subjectively through questionnaires [5,6]. Although an association between subjective and objective measures of sleep quality is expected, several studies in older people have demonstrated discrepancies between these two measures [7,8]. Moreover, previous studies have demonstrated that objective and subjective sleep quality measures have different relationships with psychological and health factors [9,10,11]. Thus, on the one hand, subjective sleep quality is positively associated with well-being and social support, and negatively associated with emotional distress, depressive symptoms, and overcommitment at work, whereas these associations were not observed with objective sleep quality [9,10]. On the other hand, worse objective, but not subjective, sleep quality is associated with higher blood pressure and hypertension [10,11]. 

Jackowska et al. [9] investigated the association between the discrepancy between subjective and objective measures of sleep quality and several psychological health factors. They observed that individuals who reported more stress at work, low levels of social support, and poor health also reported worse subjective sleep quality relative to objective sleep quality [9]. Regarding older people, previous studies indicate that, although there is an age-related deterioration in objective sleep, older individuals rate their sleep quality similarly to or higher than younger populations (e.g., [12]). Investigating the biological mechanisms behind the discrepancy between subjective and objective sleep quality is of special interest in order to understand and prevent the negative effects of sleep problems in older people.

Previous studies have demonstrated that, independently of the objective sleep quality, the activity of the Hypothalamic-Pituitary-Adrenal (HPA) axis is associated with individuals’ sleep perceptions [10,13,14,15,16,17,18]. In this context, it is particularly relevant to investigate the relationship between sleep quality and the change in cortisol levels during the night. The HPA system exhibits a clear circadian rhythm, with cortisol levels decreasing during the evening and after nocturnal sleep onset and increasing during the second half of nocturnal sleep before awakening [14,19]. Importantly, it has been indicated that a decline in the dynamic range of HPA axis activity and cortisol secretion is an indicator of dysregulation of the system [20]. Moreover, higher cortisol levels in the evening and lower levels in the morning have been associated with several health problems (e.g., [21,22]). It is well-known that cortisol has pervasive effects throughout the body and brain, impacting mood, arousal, energy, and metabolic processes, and immune and inflammatory system functioning [23]. Therefore, disruptions in cortisol secretion during the night may influence a wide variety of central and peripheral systems, and these effects cascading over the course of the night may contribute to perceptions of worse sleep quality, even when no effect on objective sleep quality is observed. Previous studies investigating changes in nighttime cortisol levels and sleep quality have shown mixed results in healthy participants and insomnia patients [24,25,26,27]. So far, no previous studies have investigated whether objective and subjective sleep quality are differentially related to changes in cortisol levels during the night in healthy older people. It is important to note that, in addition to reduced sleep quality, age has also been associated with a dysregulation of the circadian rhythm of the HPA axis. Investigating the relationship between cortisol and sleep quality may offer critical evidence about the biological mechanisms underlying age-related changes in sleep.

The aim of this study was to investigate whether individual differences in changes in cortisol levels during the night were associated with objective and subjective sleep quality and the discrepancy between the two measures in healthy older people. To do so, 45 older adults collected their cortisol levels immediately before going to bed and immediately after awakening on 90 nights (two nights per subject). Actigraphy was used to assess objective sleep quality and quantity. Questionnaires were used to assess subjective sleep quality and quantity. Following previous studies showing an association between the cortisol awakening response and subjective sleep quality (e.g., [10,16], but not objective sleep quality [10,18], we hypothesized that the change in cortisol levels from bed time until sleep termination would be related to subjective sleep quality, but not to the objective measure. Moreover, Jackowska et al. [9] showed that the discrepancy between the objective and subjective measures was related to poor health. Given that a decline in the dynamic range of HPA axis activity and cortisol secretion is an indicator of dysregulation of the system [20,21,22], we hypothesized that individuals who showed smaller changes in nighttime cortisol would rate their subjective sleep quality (measured using questionnaire) as worse than what was indicated by the objective measure of sleep quality (measured using actigraphy).

## 2. Method

### 2.1. Participants

The sample in this study was composed of 45 people from 56 to 75 years of age (men = 23, women = 22). They were volunteers recruited at university courses and seminars offered by the University of Valencia for people over 55 years old. Their participation was voluntary, they did not participate in the current research as part of the study program, and they were not consecutively enrolled. After volunteers enrolled in the study, they were interviewed to assess whether they met the inclusion criteria. Exclusion criteria were: smoking more than 10 cigarettes a day, alcohol or other drug abuse, visual or hearing problems, diabetes, presence of an HPA axis, neurological, or psychiatric disease, using any medication directly related to emotional or cognitive functioning or able to influence hormonal levels and sleep (e.g., such as glucocorticoids, psychotropic substances, or sleep medications), having been under general anesthesia in the past three months, and the presence of a stressful life event in the past year. All female participants were postmenopausal, and they had their last menstrual period at least one year before the testing time. Results on the Spanish version of the Mini-Mental Status Examination [28] indicated the absence of cognitive impairment in all participants.

### 2.2. Procedure

The study was conducted in accordance with the Declaration of Helsinki, and the protocol was approved by the Research Ethics Committee of the University of Valencia (Principal Investigator: Prof. Dr. Alicia Salvador). All participants provided written informed consent.

Participants were asked to collect salivary samples at home to measure cortisol levels immediately before going to sleep (bedtime cortisol) and immediately after awakening (waking cortisol) on two consecutive weekdays. In addition, at these two time points, participants were asked to fill in a sleep diary and questionnaire to assess subjective sleep parameters and stress perception. During salivary collection at home, the participants wore an Actiwatch 2 (Phillips Respironics, Bend, Oregon) to measure objective sleep parameters and assess the delay in collecting the first salivary sample after awakening. Moreover, in our laboratory, they completed a demographic questionnaire, the Beck Depression Inventory (BDI; [29]), to measure depressive symptomatology, and the 14-item version of the Perceived Stress Scale (PSS; [30]) to assess perceived stress in the previous month.

### 2.3. Cortisol Measurements.

The saliva samples were collected using salivettes (Sarstedt, Nümbrecht, Germany). To measure cortisol levels, salivary samples were analyzed in duplicate with the salivary cortisol enzymeimmunoassay kit from Salimetrics (Newmarket, UK). Assay sensitivity was 0.007 µg/dL, and the intra- and inter-assay variation coefficients were below 10%.

Participants were thoroughly instructed about how to provide saliva samples, and they were given written instructions. They were instructed to drink only water and not eat or brush their teeth at least 1 h prior to each saliva sample. To objectively verify participant adherence to the saliva sampling time at home, salivettes were stored in Medication Event Monitoring System (MEMS) TrackCap containers (MEMS 6 TrackCap Monitor, Aardex Ltd., Switzerland), and participants recorded the time of each saliva collection on a log.

### 2.4. Measurements of Subjective Sleep and Stress Perception at Home

Subjective sleep was assessed using a questionnaire developed by Hita-Yanez et al. [31]. Participants were asked to record the time they went to bed and their awakening time on a log. Additionally, immediately after awakening, they reported how they had slept (subjective sleep quality) using a five-point Likert scale ranging from 1 (very good) to 5 (very bad). The scale also included four other sleep-related questions. As described by the authors of the scale, the other questions assessed sleep quantity and sleep symptoms (e.g., latency to sleep onset and sleep arousals and/or wake ups after sleep onset, latency to sleep onset, sleep duration). Given that our research question is related to sleep quality, we focused on the question “how did you sleep?”, a question specifically measuring subjective overall sleep quality. Importantly, this question coincides with the definition of subjective sleep quality proposed by Ohayon et al. [6] in the first report of the National Sleep Foundation’s sleep quality recommendations. Moreover, because stress may affect cortisol levels, participants also rated how stressful they perceived the day before to be (perceived stress past day) and the stressfulness expected for the coming day (perceived stress coming day) on a five-point Likert scale ranging from 1 (not at all) to 5 (too much).

### 2.5. Objective Sleep Measurements

Objective sleep quality was assessed using the Actiwatch 2, which participants wore on their nondominant wrist during the two nights when the salivary samples were collected. The actigraphy data, recorded by the Actiwatch 2 every 15 s, were scored as asleep or awake using a medium threshold (40 activity counts) and 5-min immobility parameter to calculate sleep onset and offset. The use of actigraphy has been validated as a reliable method to assess objective sleep parameters in healthy older people [6]. It measures the degree and intensity of motion by means of a multidirectional piezoelectric accelerometer. Following the National Sleep Foundation [6], sleep efficiency (defined as the percentage of time spent asleep during the sleep period) was computed as a measure of objective sleep quality. Sleep duration was computed by subtracting the total amount of sleep measured in minutes from total time spent in bed. The actigraphy data were visually explored and edited before being processed by the scoring program. To do so, the scorer used the diary self-reports of bed and wake times to confirm the time when the participants reported going to bed and sleeping. The validated algorithm from the Respironics Actiware 5 (v.5.71, Phillips Electronics, Bend, Oregon) software was used to compute sleep efficiency and total sleep time. To identify equipment malfunction, the objective indicators of sleep quantity were validated with diary self-reports.

### 2.6. Statistical Analysis and Data Management

Cortisol values were log transformed because they did not show normal distributions. To investigate the association between cortisol and the discrepancy between subjective and objective sleep quality, we computed the sleep discrepancy index as proposed by Jackowska et al. [9]. It is important to note that the self-reported sleep quality score was assessed using a five-point Likert scale. The use of a Likert scale allowed us to assume that the intervals between the five points were equally spaced. Therefore, we treated this variable as numerical. Taking this into account, to calculate the sleep discrepancy index, we first computed the Z-scores of the measures of self-reported sleep quality and objective sleep efficiency. Then, the sleep discrepancy index was computed as the z-scored self-reported sleep quality minus the z-scored objective sleep efficiency. This index reflected the extent to which self-reported sleep quality matched objective sleep quality. Positive values indicated that individuals reported better subjective sleep quality relative to objective sleep quality. As a measure of the change in cortisol levels from bedtime to awakening (Cortisol_change_), we calculated the delta score for the cortisol levels at bedtime and immediately after awakening (cortisol at awakening minus cortisol immediately before sleep).

A linear mixed model was used to investigate the changes in cortisol levels during the night with time (bedtime and awakening) as the within-subject factor, and subject and day as random effects. Additionally, linear mixed models were performed to investigate the relationship between cortisol and sleep quality measures. Cortisol_change_ was included as the predictor (fixed factor), and subject and day were included as random effects. A restricted maximum likelihood method was used in these analyses. The use linear mixed models with the data of every night (*n* = 76), instead of aggregating the data of the two days for each participant, allowed us to obtain higher statistical power in our analyses. Moreover, by including subject and day as random effects in our models, we accounted for the nested nature of the data (i.e., sleep parameters were unlikely to be completely independent from the day before and within-participants). To investigate whether the same results were observed after controlling for other important confounders, we repeated the analyses including the following as covariates: Age, sex (0 = Men, 1 = Women), subjective socioeconomic status (measured using the nine-rung ‘social ladder’, cf., [32]), physical activity (0 = None; 1 = Low; 2 = Moderate; 3 = High), perceived stress the day before (from 1 = Not at all, to 5 = Too much), stressfulness expected for the coming day (from 1 = Not at all, to 5 = Too much), perceived stress in the previous month (score in the PSS), and depression symptoms (score in the BDI).

Salivary samples were collected by 45 participants on two consecutive nights, resulting in a total of 90 nights. The Cortisol_change_ from both nights for each participant was included in the analyses. Thirteen nights were excluded from the analyses due to missing data for cortisol (*n* = 11) or sleep quality (*n* = 2). We also screened our data for univariate and multivariate outliers (|z| ≥ 3SD). The data from one sampling night were excluded from the analyses with objective sleep quality and the discrepancy index because the values were lower than -3SD. No multivariate outliers were found in this study. The final sample included in the analyses consisted of 76 nights (44 participants; two nights from 31 participants, and one night from 13 participants). Table 1 shows the characteristics of the study sample included in the analyses.

All the analyses were performed using SPSS 24.0 (IBM SPSS Statistics 24.0, IBM, Armonk, NY, USA). All *p*-Values reported are two-tailed.

## 3. Results

### 3.1. Cortisol Levels

**Figure 1:**
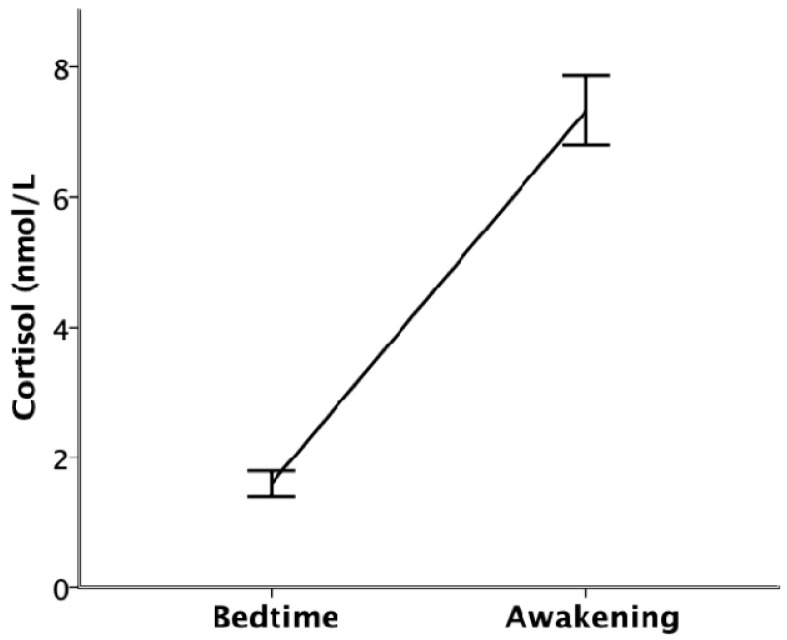
Cortisol levels (nmol/L) at bedtime and immediately after awakening. Error bars represent standard error.

The mixed-model ANOVA showed that, following the nocturnal circadian rhythm of the HPA axis, cortisol levels were significantly lower immediately before going to bed than immediately after awakening (*F*(1,147.123) = 262.133, *p* < 0.001; see Figure 1).

### 3.2. Relationship between Sleep Parameters and Cortisol

#### 3.2.1. Relationship with Subjective and Objective Sleep Quality

The results of the linear mixed-model regressions showed that higher Cortisol_change_ was significantly associated with better subjective sleep quality (*F*(1,74) = 12.896, *p* = 0.001, 95% CI [0.167, 0.589], β = 0.379), but not with objective sleep quality (*F*(1,74) = 2.179, *p* = 0.144, 95% CI [−0.306, 0.046], β = −0.130).

Importantly, the results of the linear mixed-model regressions, controlling for all the covariates, showed that higher Cortisol_change_ was still significantly associated with better subjective sleep quality (*F*(1,66) = 5.070, *p* = 0.029, 95% CI [0.028, 0.499], β = 0.263), but not with objective sleep quality (*F*(1,65) = 2.217, *p* = 0.728, 95% CI [−0.137, 0.195], β = 0.029) when the effect of important confounders was controlled in the analyses (see Table 2).

#### 3.2.2. Relationship with the Discrepancy between Subjective and Objective Sleep Quality

To investigate whether Cortisol_change_ was associated with the discrepancy between the measures of subjective and objective sleep quality, we repeated these analyses, including the sleep discrepancy index as the dependent variable and Cortisol_change_ as the predictor. The results showed a significant association between higher Cortisol_change_ and a higher sleep discrepancy index when the analyses were performed to control for sleep quantity (*F*(1,73) = 13.164, *p* = 0.001, 95% CI [0.163, 0.560], β = 0.361) and when the analyses were performed to control for all the other covariates (i.e., age, sex, subjective socioeconomic status, physical activity, perceived stress the day before, stressfulness expected for the coming day, perceived stress in the previous month, and depression symptoms) (*F*(1,65) = 7.073, *p* = 0.010, 95% CI [0.072, 0.507], β = 0.290) (see Table 3).

#### 3.2.3. Analyses Excluding Participants Suspected of Nonadherence to the Protocol

Given that a delay in the first salivary sample immediately after awakening affects the reliability of the measurement of the first salivary sample due to the beginning of the cortisol awakening response [33], the information provided by the MEMS TrackCap containers, the Actiwatch, and the participant’s log were crossmatched to confirm waking and salivary collection times. Following Stalder et al. [33], study days with a difference between awakening and saliva collection greater than 5 min were excluded from the analyses. The final number of nights included in the analyses was 67. As observed with the complete sample, these results showed that higher Cortisol_change_ was related to better subjective sleep quality (Controlling for sleep quantity: (*F*(1,64) = 16.234, *p* < 0.001, 95% CI [0.228, 0.678], β = 0.453); controlling for all the covariates: (*F*(1,56) = 7.655, *p* = 0.008, 95% CI [0.082, 0.576], β = 0.334)) and higher sleep discrepancy index (Controlling for sleep quantity: (*F*(1,64) = 15.771, *p* < 0.001, 95% CI [0.209, 0.632], β = 0.421); controlling for all the covariates: (*F*(1,56) = 9.600, *p* = 0.003, 95% CI [0.123, 0.571], β = 0.347)). Cortisol_change_ was not related to objective sleep quality (Controlling for sleep quantity: (*F*(1,64) = 1.817, *p* = 0.182, 95%CI [−0.323, 0.063], β = −0.130); controlling for all the covariates: (*F*(1,56) = 2.229, *p* = 0.141, 95%CI [−0.344, 0.050], β = −0.147)). Therefore, the statistical conclusions of the study remained the same after participants suspected of nonadherence to the protocol were excluded from the analyses.

## 4. Discussion

We observed that larger changes in cortisol levels between sleep onset and awakening, reflecting a healthier circadian rhythm of the HPA axis, were associated with better subjective sleep quality, but not with objective sleep quality. Moreover, smaller changes in nighttime cortisol were associated with an underestimation of sleep quality. All these results were observed even after controlling for important confounders, such as sleep quantity, age, sex, subjective socioeconomic status, stress perception, depression, and physical activity, and after excluding participants suspected of nonadherence to the protocol from the analyses.

Larger changes in cortisol levels, reflecting a healthier circadian rhythm of the HPA axis, were associated with better subjective sleep quality, but not with objective sleep quality. It is well-known that cortisol secretion, the end product of the HPA axis, is associated with the sleep–wake cycle [13]. Previous studies have reported different relationships between cortisol and measures of objective and subjective sleep quality. For instance, the cortisol awakening response, a marker of HPA axis activity closely related to arousal and termination of sleep inertia [14], has been negatively associated with subjective sleep quality (e.g., [10,15,16]; but see [17]), but not with objective measures [10,18]. Thus, the current study supports previous research showing a relationship between subjective sleep quality and the activity of the HPA axis. Moreover, our results agree with previous studies showing a lack of relationship between changes in cortisol levels during the night and objective sleep quality [25,26,27]. Together, these observations indicate that individuals with a healthier circadian rhythm of the HPA axis would report better subjective sleep quality. Within this context, previous studies have demonstrated that reduced sleep quality is associated with several age-related health problems, such as cognitive deficits and cardiovascular diseases [2,3,4], and that these health problems have also been associated with a dysregulation of the circadian activity of the HPA axis (e.g., [34,35,36]. Based on our results, it is possible that these health problems associated with poorer sleep quality in older people are due to a dysregulation of the HPA axis.

This study also shows, for the first time, that interindividual differences in the change in cortisol levels during the night are associated with the discrepancy between measures of subjective and objective sleep quality. This result is consistent with previous research showing a similar association between this index and worse health factors, such as stress at work, low levels of social support, and poor health [9]. Although it is not possible to interpret the meaning of the values on the sleep discrepancy index (i.e., the range of values that can be considered “normal” or “healthy” is not known), the current evidence indicates that individuals reporting worse subjective sleep quality relative to objective sleep quality also present worse psychological and biological health factors. More research with larger samples and longitudinal studies are still needed to increase our knowledge about this index.

The specific association between cortisol and both subjective sleep quality and the discrepancy in sleep quality is of interest. A possible explanation for these results is related to the fact that the activity of the HPA axis affects several central and peripheral systems, such as metabolic processes and the immune and inflammatory systems, and it can affect mood, arousal, and energy [23]. The acute response of the HPA axis and its influence are especially important during stressful situations [37]. However, this response may also affect central and peripheral systems during both the day and night under low stress conditions. Thus, a dysregulation of the HPA axis could provoke acute increases in arousal or reactions of metabolic systems during the night. These disruptions in cortisol secretion during the night may contribute to *perceptions* of worse sleep quality. Considering the lack of relationship between cortisol and actigraphy data, these results indicate that the influence of the HPA axis would occur even when objective sleep quality is not affected. Further longitudinal studies are needed to establish a causal pathway between changes in variables associated with a dysregulation of the HPA axis and changes in sleep quality via nighttime HPA axis activity.

Although it was not the focus of this study, previous studies have shown that subjective sleep quality is associated with stress at work [9]. In our study, however, we did not observe significant relationships between subjective sleep quality and stress measures. In contrast, worse objective sleep quality was significantly related to more stress expected for the upcoming day and marginally related to perceived stress during the previous month. The differences in the study samples may explain the discrepancy in our results. Jackowska et al. [9] recruited women younger than 45 years old. In our study, all the participants were above 55 years old, and most of them were retired. These observations highlight the importance of future studies investigating age-related differences in the relationship between psychological and endocrine stress measures and sleep parameters.

Despite the novel findings of this study, some limitations should be considered. In order to control for possible cofounders, our study did not include participants with sleep problems or other common age-related health issues. Therefore, the results may not be generalized to other populations. Moreover, although it has been demonstrated that the Actiwatch 2 is a valid method to assess sleep quality in older people [38], it does not provide information about sleep phases or micro awakenings. The use of polysomnography, the gold standard for sleep assessment, may provide important information in future studies. Also related to the assessment of sleep quality, in this study, we used sleep efficiency as a marker of objective sleep quality. The selection of this index was based on the National Sleep Foundation’s sleep quality recommendations. The Sleep Quality Consensus panel of this foundation concluded that sleep efficiency is a good marker of sleep quality across all age groups [6]. Moreover, previous research has shown that, in contrast to other sleep parameters, sleep efficiency continues to significantly decline in older populations [39]. This is of special interest for the current study given our study sample. However, it is important to note that there are other sleep parameters that reflect sleep quality and can be computed from actigraphy and polysomnography recordings. Future studies may benefit from using more advanced analysis of the actigraphy signal or polysomnography recordings when investigating the association between cortisol and objective sleep quality. Finally, given the cross-sectional nature of this study, we cannot exclude the possibility that differences in sleep quality explain the changes in HPA axis activity.

## 5. Conclusions

In conclusion, interindividual differences in nighttime cortisol secretion were associated with differences in self-reported measures of sleep quality, but not with objective measures. Moreover, individuals with worse regulation of the HPA axis reported worse subjective sleep quality relative than objective sleep quality. This study demonstrates that individual differences in sleep quality in older people may be explained, to some extent, by the activity of the HPA axis.

## Figures and Tables

**Table 1:** Characteristics of the sample.

Variables	Mean	SD
Age (years)	63.89	4.06
SES	5.62	1.27
Physical activity	1.78	0.77
Education	3.76	1.30
PSS	18.60	5.77
BDI	5.04	4.46
Objective sleep time (min)	333.44	57.48
Subjective sleep time (min)	400.53	63.03
Objective sleep quality (Efficiency)	81.19	10.13
Subjective sleep quality	3.53	0.92
Perceived stress day before	2.05	0.92
Stress expected coming day	2.17	0.82
Cortisol_change_ (nmol/L)	1.63	0.72

Note: *n* = 44; 76 nights. SES = Subjective socioeconomic status; PSS = Perceived Stress Scale; BDI= Beck Depression Inventory. Physical activity: From 0 (none) to 3 (high); Education: 0 (no studies), 1 (primary school), 2 (secondary education), 3 (bachelor’s and master education), 4 (doctorate); Subjective sleep quality: From 1 (very good) to 5 (very bad); Perceived stress the day before: From 1 (not at all) to 5 (too much); Stressfulness expected for the coming day: From 1 (not at all) to 5 (too much).

**Table 2:** Relationship between Cortisol_change_ and subjective and objective sleep quality.

Variables	β	*p*	95% Confidence Interval	
DV: Subjective Sleep Quality				
Cortisol_change_ (nmol/L)	0.263	0.029	[0.028, 0.499]	
Objective sleep time (min)	0.169	0.155	[−0.066, 0.404]	
Age (years)	−0.097	0.435	[−0.342, 0.149]	
SES	−0.058	0.627	[−0.293, 0.178]	
Physical activity	0.068	0.561	[−0.164, 0.300]	
Sex	−0.127	0.329	[−0.386, 0.132]	
Perceived stress day before	−0.191	0.132	[−0.441, 0.059]	
Stress expected coming day	0.162	0.209	[−0.093, 0.416]	
PSS	0.122	0.337	[−0.130, 0.375]	
BDI	−0.124	0.385	[−0.406, 0.159]	
DV: Objective sleep quality				
Cortisol_change_ (nmol/L)	0.029	0.728	[−0.137, 0.195]	
Objective sleep time (min)	−0.138	0.141	[−0.323, 0.047]	
Age (years)	0.586	0.000	[0.401, 0.771]	
SES	0.166	0.090	[−0.027, 0.359]	
Physical activity	−0.119	0.204	[−0.304, 0.066]	
Sex	0.134	0.147	[−0.048, 0.317]	
Perceived stress day before	0.153	0.139	[−0.051, 0.356]	
Stress expected coming day	−0.229	0.023	[−0.426, −0.032]	
PSS	0.200	0.050	[0.000, 0.401]	
BDI	−0.078	0.433	[−0.276, 0.120]	

Note: *n* = 44; 76 nights. SES = Subjective socioeconomic status; PSS = Perceived Stress Scale; BDI = Beck Depression Inventory. Objective sleep time (min) was obtained from sleep duration computed from actigraphy.

**Table 3:** Relationship between Cortisol_change_ and the discrepancy between subjective and objective sleep quality.

Variables	β	*p*	95% Confidence Interval	
DV: Discrepancy in sleep quality				
Cortisol_change_ (nmol/L)	0.290	0.010	[0.072, 0.507]	
Objective sleep time (min)	−0.301	0.007	[−0.518, −0.084]	
Age (years)	−0.190	0.099	[−0.416, 0.037]	
SES	0.044	0.686	[−0.173, 0.262]	
Physical activity	−0.048	0.657	[−0.263, 0.167]	
Sex	−0.202	0.097	[−0.441, 0.037]	
Perceived stress day before	0.027	0.814	[−0.204, 0.258]	
Stress expected coming day	−0.028	0.814	[−0.263, 0.207]	
PSS	0.144	0.219	[−0.088, 0.377]	
BDI	−0.024	0.854	[−0.285, 0.237]	

Note: *n* = 44; 76 nights. SES = Subjective socioeconomic status; PSS = Perceived Stress Scale; BDI = Beck Depression Inventory. Objective sleep time (min) was obtained from sleep duration computed from actigraphy.
